# Muscle preservation during hospitalization: energy balance, protein intake, and habitual physical activity

**DOI:** 10.1097/MCO.0000000000001154

**Published:** 2025-08-13

**Authors:** Cas J. Fuchs, Luc J.C. van Loon

**Affiliations:** Department of Human Biology, Institute of Nutrition and Translational Research in Metabolism (NUTRIM), Maastricht University Medical Centre^+^, Maastricht, the Netherlands

**Keywords:** bed rest, exercise mimetics, muscle atrophy, muscle mass, muscle protein synthesis

## Abstract

**Purpose of review:**

Muscle loss during hospitalization is a major clinical concern, as it has been associated with reduced physical function, quality of life, and increased mortality. This review outlines the key causes of muscle wasting and highlights practical strategies to support muscle mass preservation during hospitalization.

**Recent findings:**

Physical inactivity, along with reduced energy and protein intake, are the primary drivers of muscle atrophy during hospitalization by suppressing muscle protein synthesis (MPS). Maintaining energy balance is critical to prevent declines in MPS rates and attenuate muscle loss. Preserving habitual protein intake is essential and, when total energy intake is reduced, should be achieved through a more protein-dense diet. Preventing disuse atrophy requires at least some level of daily physical activity. Physical activity sensitizes skeletal muscle to the anabolic properties of protein ingestion, enabling greater use of protein-derived amino acids for MPS. Therefore, frequent in-hospital movements, such as bed-to-chair transfers and walking, should be encouraged. When voluntary activity or muscle contractions are impossible, exercise mimetics, like neuromuscular electrical stimulation, may be applied to stimulate muscle activity and limit muscle mass loss.

**Summary:**

Preserving muscle mass during hospitalization requires a multimodal approach: achieving energy balance, maintaining protein intake, minimizing muscle disuse, and, whenever necessary, apply exercise mimetics.

## INTRODUCTION

Hospitalization leads to rapid and substantial muscle loss. In otherwise healthy young individuals, short-term bed rest results in a loss of approximately 0.5% of leg muscle mass per day [1]. During critical illness, this process accelerates dramatically with reports showing that patients may lose nearly 3% of leg muscle mass per day in the ICU [2]. Earlier work by our laboratory [3] and others [4] similarly demonstrated that up to 15% or more of leg muscle mass can be lost within just 1 week in critically ill or comatose patients. Collectively, these findings underscore not only the early onset of disuse atrophy but also its rapid progression to acute muscle wasting and the potential development of sarcopenia, particularly in critically ill patients. Within a hospital duration of 4–44 days, ~20% of hospitalized patients develop sarcopenia, with the highest incidence (~60%) observed among trauma patients in the ICU [5]. These numbers are not only concerning in terms of prevalence but also in light of the significant clinical consequences associated with in-hospital muscle loss.

**FIGURE 1:**
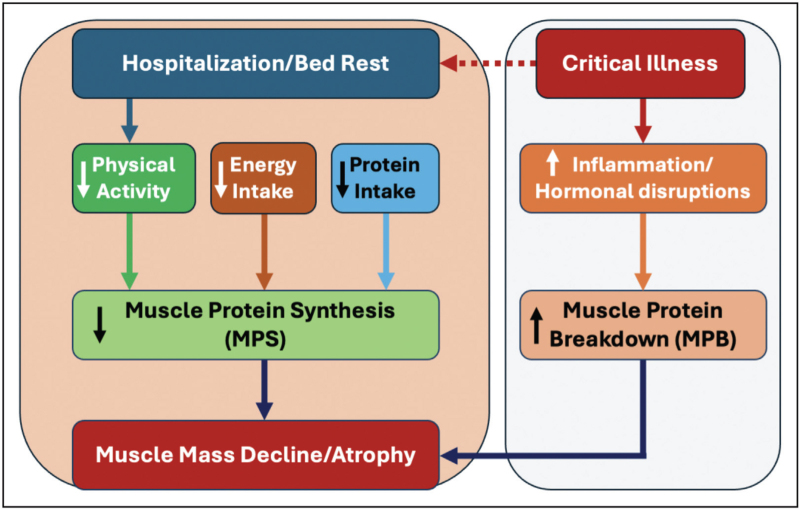
Key mechanisms driving muscle loss during hospitalization. Hospitalization and bed rest lead to decreased physical activity, resulting in reduced basal and postprandial muscle protein synthesis (MPS). In critically ill patients, muscle protein breakdown (MPB) may also be elevated, at least partly, due to high levels of systemic inflammation and hormonal disruptions, further accelerating muscle loss. In parallel, reduced energy and protein intake – common in hospitalized settings – exacerbate anabolic resistance and limit the availability of substrates needed for MPS. Together, these factors lead to a rapid decline in muscle mass.

Recent studies have shown that critically ill patients with reduced muscle mass require mechanical ventilation more frequently and for longer durations, have higher rates of tracheotomy, and experience prolonged ICU stays [6]. In addition, low muscle mass is associated with reduced quality of life, increased risk of falls and fractures, and, ultimately, a substantially elevated mortality rate [7–9]. These findings highlight not only the clinical severity but also the economic and social burden of in-hospital muscle loss, reinforcing the urgent need for effective interventions to preserve muscle mass during periods of bed rest. However, before such strategies can be developed, a deeper understanding of the underlying mechanisms of muscle loss during hospitalization is essential (Fig. 1). 

**Box 1:**
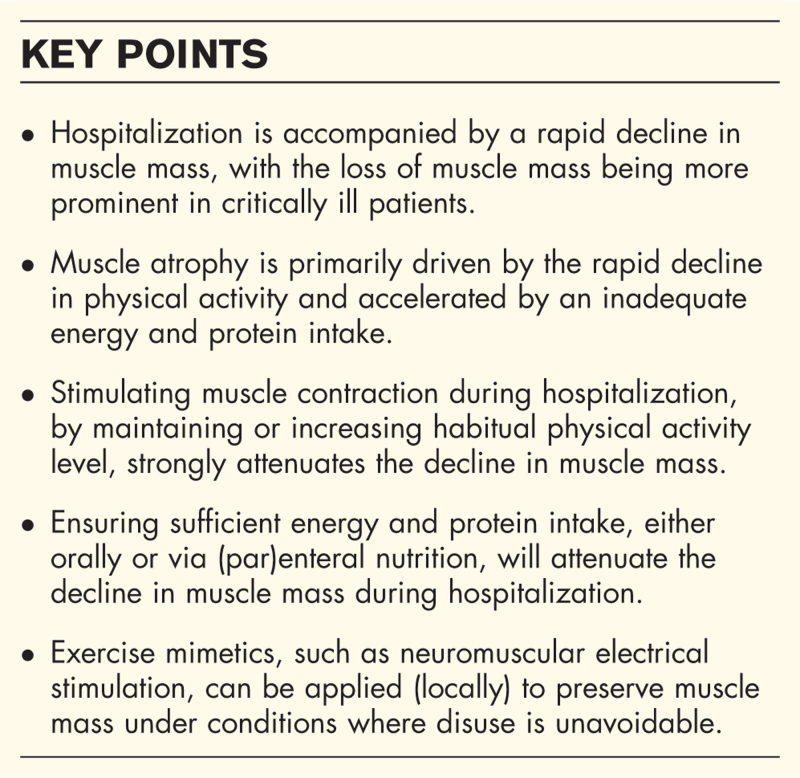
no caption available

Skeletal muscle mass is maintained through a dynamic balance between muscle protein synthesis (MPS) and muscle protein breakdown (MPB), with approximately 1–2% of muscle proteins being synthesized and degraded each day [10]. Physical activity and food intake acutely stimulate MPS, thereby supporting the maintenance of skeletal muscle mass under typical ambulatory and healthy conditions. However, during periods of hospitalization, this balance is disrupted. Physical activity is substantially reduced or even absent because of bed rest. As a result, lower rates of MPS are typically observed in bedridden individuals [11,12^▪▪^]. More specifically, both a rapid decline in basal MPS and the development of anabolic resistance, an impaired muscle anabolic response to protein intake, have been reported in response to short-term muscle inactivity [13].

In addition to reduced/absent physical activity, food intake is often compromised in hospitalized patients, particularly in those who are critically ill. The resulting energy restriction suppresses basal MPS and induces anabolic resistance [14]. In otherwise healthy individuals, even a modest energy deficit of ~20% for 10 days has already been shown to reduce MPS by ~20% [15]. Consequently, a negative energy balance exacerbates muscle loss. Recent work has shown a correlation between lower caloric intake and greater muscle atrophy within just the first 3 days of ICU admission [7]. The lower energy intake is typically accompanied with a lower protein intake, further compromising postprandial protein synthesis rates and exacerbating muscle mass loss. Daily protein intake in hospitalized patients is generally well below the recommended protein intake of 0.8 g/kg body mass/day for healthy adults, and does not even come close to guidelines (1.2–1.5 g/kg body mass/day) for clinically compromised patients [16^▪^]. In support, lower protein intakes have been associated with greater muscle atrophy in patients admitted to the ICU [7]. In addition to compromised MPS, MPB may also be elevated during hospitalization, particularly in critically ill patients [4,17]. This increase may be, at least partly, driven by high levels of systemic inflammation and hormonal disruptions associated with critical illness. However, MPB does not seem to be elevated in otherwise healthy bedridden individuals [11,18], implying that the decline in basal and postprandial MPS play a more dominant role in the observed loss of muscle mass in most hospitalized patients.

Given the detrimental impact of reduced physical activity and inadequate energy and/or protein intake, preserving muscle mass through targeted interventions, such as optimized nutritional strategies and early mobilization or even prehabilitation, is essential for improving patient outcomes and reducing hospitalization-related complications.

## MAINTAINING ENERGY BALANCE AND HABITUAL PROTEIN INTAKE

To attenuate the decline in basal and postprandial MPS, it is of importance to maintain energy balance and preserve habitual protein intake levels during hospitalization. Although achieving perfect energy balance can be challenging, it will be important to aim for, as – in addition to the detrimental impact of energy deficiency – overfeeding during prolonged bed rest may increase fat mass and could also exacerbate muscle loss [19,20]. Therefore, energy needs should be assessed using indirect calorimetry whenever possible, given the substantial daily variation within and between (critically ill) patients [21] and the inaccuracies associated with the applied predictive equations. Recent work suggests that nutrition guided by indirect calorimetry leads to better preservation of muscle mass and may improve short-term mortality outcomes when compared to weight-based estimations [22,23]. If indirect calorimetry is unavailable, ventilator-derived VCO_2_ or VO_2_ measurements may provide an alternative in the ICU. In the absence of these methods, simple estimations based on body mass (e.g. 20–25 kcal/kg body mass/day) may be used, although they should be interpreted with caution. Overall, a personalized approach to energy intake is recommended [21]. To maintain habitual protein intake in the light of a decline in energy intake and/or energy requirements, a more protein dense diet should be pursued. Maintaining or (slightly) increasing habitual protein intake (from 1 to up to 1.3 g/kg body mass/day) is likely sufficient [24]. Higher protein intakes of 2 g/kg body mass/day or more could lead to worse clinical outcomes, including increased gastrointestinal intolerance and impaired health-related quality of life after discharge [25^▪▪^]. Most hospitalized patients fail to reach even minimal intake levels, with average protein intake reported at ~0.7 g/kg body mass/day in general hospitalized populations [16^▪^], and as low as 0.55 g/kg body mass/day in patients in the ICU [3]. Clearly, achieving adequate protein intake is important, and therefore, strategies should be employed to increase protein intake levels in hospitalized patients.

For relatively healthy patients who are able to eat normally despite being hospitalized, increasing protein (and energy) intake through oral nutrition may be manageable, though individual tolerance varies. Recent work from our lab has demonstrated that providing an energy-dense and protein-dense snack prior to sleep can effectively enhance daily intake in this population, enabling 12% of patients to meet habitual protein intake targets [16^▪^]. However, many patients still struggle to consume larger volumes of food or additional snacks. For these individuals, regular meals may be fortified with protein isolates or concentrates or the diet could be supplemented with oral nutritional (protein-rich) supplements to achieve protein intake levels. Notably, even small doses (3.6 g) of essential amino acids may be applied to stimulate MPS [26]. Even when achieving energy balance is difficult or impossible, providing adequate absolute amounts of protein and/or amino acids remains critical. Indeed, the addition of easily digestible proteins or essential amino acids has been shown to stimulate MPS even under conditions of energy deficiency [14,27]. Collectively, these findings underscore the importance of optimizing protein and amino acid provision during hospitalization. However, more research is warranted to identify the most effective oral nutritional strategies for overcoming anabolic resistance and preserving muscle mass in clinical populations.

Whereas oral nutritional intake is preferred whenever possible, it is generally not feasible for critically ill patients (e.g. those with traumatic injury, in a comatose state, or experiencing severe metabolic stress). In these cases, nutritional support via enteral or parenteral routes becomes critical. Although parenteral feeding delivers energy and nutrients directly into the systemic circulation and is sometimes necessary (particularly when enteral nutrition is insufficient or not feasible), enteral nutrition is generally preferred, at least in patients with functional gastrointestinal systems, due to its support of gut integrity and lower complication rates [28]. When providing enteral nutrition (duodenal delivery), we have previously shown that protein digestion and amino acid absorption are not compromised in critically ill patients [29]. Furthermore, enteral provision of free amino acids as opposed to intact protein may improve systemic delivery of amino acids [30^▪^]. Collectively, even in the critically ill, it is possible to administer sufficient energy and or protein into the systemic circulation to support peripheral tissues, such as the muscle. However, despite adequate substrate availability, anabolic resistance compromises the capacity of protein feeding to stimulate MPS in critically ill patient populations [29]. This limits the effectiveness of nutritional interventions that attenuate the loss of muscle mass, highlighting the need for adjuvant strategies.

## PREVENT MUSCLE DISUSE

In addition to providing adequate nutrition, stimulating muscle contractions through maintaining or increasing physical activity forms a key pillar in preserving basal and postprandial MPS in hospitalized patients. Physical activity enhances the muscle's sensitivity to the anabolic properties of protein and amino acid administration, thereby offsetting some of the anabolic resistance [31,32]. Even a minimal level of habitual physical activity, such as transferring from the bed to a chair or walking to the bathroom, may attenuate the decline in basal and postprandial MPS and attenuate muscle loss during hospitalization. Supporting this concept, recent work has shown that performing a single bout of resistance exercise prior to a 5-day bed rest period can significantly attenuate subsequent declines in myofibrillar protein synthesis rates and preserve muscle mass throughout bed rest [12^▪▪^]. The fact that such a brief intervention exerted protective effects over several days highlights the potency of even short bouts of physical activity or exercise to mitigate muscle loss during disuse. However, the minimal effective dose, timing, and type of physical activity required to counteract anabolic resistance and preserve muscle mass during hospitalization remains to be established. Establishing this threshold is essential for developing feasible, low-intensity exercise interventions tailored to patients with limited mobility or reduced functional capacity. Such protocols could play a key role in preserving muscle health and improving recovery outcomes during and after hospitalization. Indeed, increasing attention has been directed towards evaluating the benefits of physical activity during hospitalization, with recent research demonstrating that in-hospital multicomponent exercise programs can significantly improve both physical performance and even cognitive function [33].

It is important to recognize, however, that not all patients, such as those who are comatose or experiencing severe functional limitations, are able to perform habitual daily living activities or low-intensity exercise. In these cases, alternative (passive) strategies to stimulate MPS and prevent muscle atrophy may be considered.

## EXERCISE MIMETICS

Some patients are unable to engage in active movement, and passive mobilization is generally used as part of standard care to maintain joint range of motion and prevent stiffness and excessive fibrosis. However, this is insufficient to prevent skeletal muscle wasting. Therefore, muscle contractions should be actively induced through alternative strategies to help preserve muscle mass. Among the available options, neuromuscular electrical stimulation (NMES) is currently the most established and clinically feasible approach to counteract (local) disuse atrophy. The application of NMES has been shown to increase MPS and may help attenuate muscle loss in immobilized or critically ill patients [3,34^▪^]. In previous work from our laboratory, twice daily application of NMES prevented skeletal muscle atrophy during ~7 days of hospitalization in fully sedated patients [3]. Beyond NMES, additional strategies aimed at inducing anabolic effects in the absence of voluntary movement are being explored. One area of interest is blood flow restriction (BFR), which initially appeared promising. However, in the absence of muscle contractions, its efficacy to preserve muscle mass does not seem effective in a setting of disuse [11,35]. These findings emphasize the importance of continuing to explore innovative exercise mimetics that can be applied safely and effectively in patients unable to participate in traditional physical activity or in patients with limb immobilization. In particular, it will be crucial to identify strategies that also attenuate disuse-induced declines in muscle strength, as current approaches (e.g. NMES) have limited impact on preserving muscle function. Further research is warranted to refine these approaches and develop evidence-based protocols for muscle preservation that can be integrated into clinical care.

## PREHABILITATION

Hospitalization due to disease or trauma is often unpredictable and, as such, strategies to mitigate muscle loss are typically implemented throughout admission and/or subsequent postdischarge rehabilitation [18,36]. In case of elective surgery, it is possible to apply prehabilitation measures as a potential strategy to enhance physical reserves and improve patient outcomes during subsequent hospitalization. Given that muscle can be lost up to five times faster than it is gained [37,38], increasing muscle mass and function prior to hospitalization may be of benefit to the patients’ needs. Indeed, as noted earlier, even a single session of resistance exercise prior to disuse has been shown to attenuate the decline in MPS and attenuate some muscle mass loss [12^▪▪^]. Therefore, in the context of planned hospitalizations, prehabilitation should be actively encouraged as a proactive strategy to preserve muscle health and accelerate subsequent recovery.

## CONCLUSION

**FIGURE 2:**
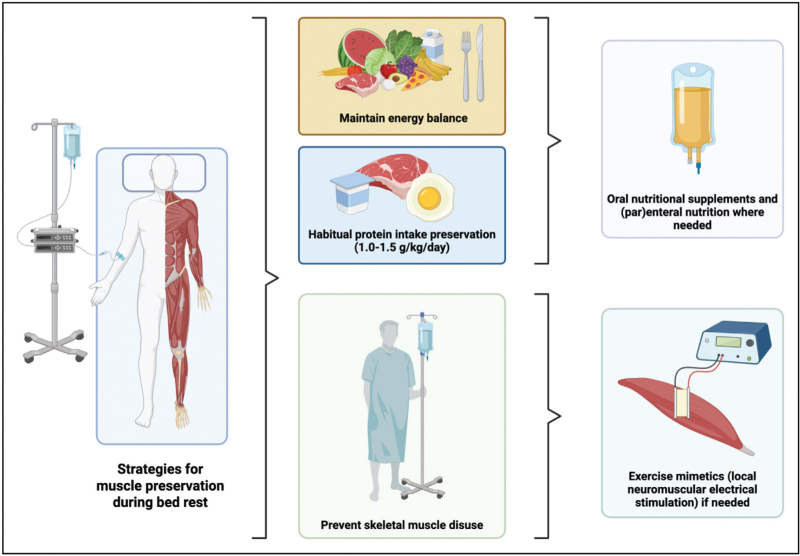
Multimodal strategies are required to preserve muscle mass during hospitalization. Created with BioRender.com.

Muscle preservation during hospitalization requires a multifaceted approach that targets the primary drivers of muscle loss: inadequate food intake and physical inactivity (Fig. 2).

Key strategies include:

Energy balance: energy deficits can accelerate muscle atrophy. Patients should be stimulated to maintain their caloric intake during hospitalization.

Protein intake: many hospitalized patients consume far less protein than generally recommended. A decline in habitual protein intake accelerates disuse atrophy and should be prevented. Patients should aim to consume 1–1.5 g protein/kg body mass/day.

Physical activity: muscle loss during hospitalization is largely attributed to a substantial decline in physical activity level. Maintaining or increasing physical activity level enhances the muscle's sensitivity to protein and amino acid intake and attenuates the decline in muscle mass.

Alternative strategies: for patients (partly) unable to engage in voluntary physical activity (e.g. those in the ICU or in comatose states) exercise mimetics, such as NMES, could be considered to allow (local) muscle contractions and preserve muscle mass.

## Acknowledgements

*The authors acknowledge the valuable contributions of other authors in the field who were not cited in this review due to the journal's requirement to prioritize references from the past 18 months.*

### Financial support and sponsorship

*None.*

### Conflicts of interest

*C.J.F. and L.J.C.v.L. have no conflicts of interest related to this work. L.J.C.v.L. and his laboratory have received research grants, consulting fees, speaking honoraria, or a combination of these for research on the impact of exercise and nutrition on muscle metabolism. A full overview of research funding is provided at https://www.maastrichtuniversity.nl/ljc-van-loon.*

## REFERENCES AND RECOMMENDED READING

Papers of particular interest, published within the annual period of review, have been highlighted as:

▪ of special interest
▪▪ of outstanding interest
